# The regulatory effects of maize saving irrigation in arid region

**DOI:** 10.3389/fpls.2025.1641434

**Published:** 2025-08-29

**Authors:** Yuhao Zhao, Xin Li, Meiwei Lin, Chen Gao, Xiaoya Li, Kunkun Wu, Liang He, Weihong Sun

**Affiliations:** ^1^ School of Agricultural Engineering, Jiangsu University, Zhenjiang, China; ^2^ School of Computer Science and Technology, Xinjiang University, Urumqi, China; ^3^ Department of Electronic Engineering, and Beijing National Research Center for Information Science and Technology, Tsinghua University, Beijing, China

**Keywords:** arid region, regulated deficit irrigation, maize, physiological responses, yield and quality, TOPSIS-entropy weight method

## Abstract

The increased drought stress caused by worldwide climate-change-driven aridification has exacerbated water scarcity in agricultural production, posing a significant challenge to agricultural sustainability. This study was conducted at Huaxing Farm in Changji City, Xinjiang, establishing five irrigation gradients: 100% (CK), 90% (W1), 80% (W2), 70% (W3), and 60% (W4) of the conventional water supply (full irrigation requirement). The primary objective was to systematically investigate the effects of water regulation on physiological and biochemical parameters, yield formation, and kernel quality in maize plants. A multidimensional TOPSIS-entropy weight method was used to evaluate the effectiveness of these irrigation treatments in the context of drought adaptation. The results indicated that moderate regulated deficit irrigation (W1) increased yield by 8.0% while using 10% less water. This treatment also led to higher protein levels (7.59g/100g) and starch content (68.1g/100g). In contrast, severe regulated deficit irrigation (W4) failed to alleviate drought stress, which significantly induced biomass loss and inhibited yield formation. A comprehensive review revealed that W1 was the top-performing treatment, achieving the highest overall evaluation index of 0.728. W1 activated a synergistic mechanism that combined osmotic adjustment and antioxidant defense. This specific physiological adaptation was characterized by elevated proline accumulation, activation of key enzyme systems, and stabilization of malondialdehyde levels, which indicated effective mitigation of drought-induced cellular damage. This physiological optimization improved photoassimilate partitioning to the kernels. Therefore, W1 represented a promising irrigation strategy, providing insights into the physiological basis for synergistic stress resistance triggered by moderate water deficit and enabling yield gains with 10% less irrigation.

## 1 Introduction

With the intensification of worldwide climate change, drought conditions have become increasingly severe, emerging as a critical constraint on sustainable agricultural development (Ahmad et al., 2023; Gul et al., 2023). According to the data from the Food and Agriculture Organization of the United Nations (FAO), the frequency and intensity of worldwide drought events have exhibited a marked upward trend over the past decades, leading to escalating agricultural production losses of mounting severity (Lu et al., 2019; Chen et al., 2025). Moreover, the uneven spatiotemporal distribution of water resources has posed significant challenges to agricultural irrigation in arid and semi-arid regions (Wu et al., 2024). Therefore, agricultural production now faces a pivotal challenge: achieving high-efficiency water conservation while maintaining crop yield and quality, and this issue demands urgent resolution (Darko et al., 2015; Wu et al., 2020).

Maize (Zea mays L.) is a globally significant food, forage, and industrial feedstock crop whose yield stability is directly linked to food security and the sustainable development of agricultural economies (Nuss and Tanumihardjo, 2010; Shiferaw et al., 2011). However, compared to other dry grain crops, maize is characterized by high water demands and acute sensitivity to water stress. Maize plants face particular vulnerability during the tasseling to filling stages—a critical drought-sensitive period. Water stress during this phase directly induces yield losses ranging from 20% to 50% (Darko et al., 2015; Sheoran et al., 2022; Hu et al., 2023). Therefore, establishing appropriate irrigation decision-making for crops holds profound significance for ensuring the sustainable development of maize production systems in arid and semi-arid areas.

Numerous studies have demonstrated that abiotic stress significantly disrupts physiological and biochemical processes in maize plants (Farooq et al., 2009; Khan et al., 2024). Drought stress has been shown to differentially inhibit photosynthesis, respiration, and nutrient uptake/translocation in maize plants, thereby impairing dry matter accumulation and distribution, which ultimately causes yield reduction (Hu et al., 2023; Cai et al., 2024). For instance, a study demonstrated that severe water stress-induced stomatal closure in maize leaves, restricting CO_2_ influx and causing a substantial decline in photosynthetic efficiency (Yu et al., 2015); another study revealed that drought impeded the growth and development of maize root systems, significantly reducing water and nutrient uptake capacities (Qi et al., 2010). Concurrently, research has documented that moderate water stress enhanced the contents of quality-related components (notably protein, starch, and lipids) through strategic regulation of physiological processes and metabolic pathways (Ge et al., 2010; Chen et al., 2021).

Various strategies have been developed to mitigate the impacts of drought stress, primarily involving the development of drought-resistant cultivars, optimization of water-saving irrigation technologies, plastic film mulching systems, and so on (Li and Liu, 2020; Shabbir et al., 2020; Yan et al., 2022). Regulating deficit irrigation (RDI) has emerged as an extensively implemented approach. This technique activates crop drought resistance mechanisms by managing irrigation quotas to create controlled water deficit conditions while enhancing water use efficiency (WUE) (Fereres and Soriano, 2006; Rai et al., 2022). It ensures adequate water during critical periods while applying controlled deficits at other stages, activating the crop’s self-regulating mechanisms to enhance WUE (Chai et al., 2015; Allakonon et al., 2022; Wu et al., 2022).

RDI has been shown to upregulate key transcription factors in maize, which exhibit distinct spatiotemporal expression patterns across different tissues and developmental stages. This molecular response enhances the plant’s adaptive capacity to water deficit conditions, thereby improving drought tolerance (Seeve et al., 2017). Furthermore, studies have demonstrated that RDI not only increases WUE but also stimulates root system proliferation, effectively reducing deep percolation water losses while optimizing soil water extraction (Wu et al., 2018; Zou et al., 2021). Current research has implemented RDI trials on various crops, with partial results confirming that this technique could maintain yield levels while significantly conserving water (Rasool et al., 2020; Wang et al., 2020; Zou et al., 2021). For instance, some researchers implemented RDI in rice cultivation, achieving a 31.4% yield increase with 20% less irrigation water while significantly improving grain quality (Gao et al., 2024); Cotton trials revealed that optimized RDI strategies not only increased yield but also enhanced fiber quality (Lin et al., 2024). However, although deficit irrigation can enhance maize resilience to drought stress, determining the optimal irrigation level under RDI remains a significant challenge. Current methodologies fail to provide real-time and accurate water status information, resulting in difficulties in precise irrigation management. This limitation may lead to yield penalties due to suboptimal water stress imposition. Therefore, the optimal irrigation regimes for various maize varieties, soil types, and climatic conditions have not yet been clearly defined.

The unique climatic conditions of Huaxing Farm in Changji City, Xinjiang, provide an ideal experimental environment for research on water-saving irrigation in maize cultivation. Characterized by an arid climate and scarce precipitation, this region faces prominent contradictions between water supply and crop water requirement. Under the context of intensifying global droughts, Xinjiang, as a typical arid agricultural region, holds strategic importance for investigating maize water-saving irrigation mechanisms. This study aims to evaluate the regulatory effects of RDI on maize, with a focus on its impact on physiological development, yield, and quality attributes under drought stress in Xinjiang. The collected data was thoroughly analyzed using the TOPSIS-entropy weight method to evaluate the results. This method comprehensively considered the interrelationships among various indicators and determined the indicator weights objectively, finally identifying the optimal irrigation scheme which accurately suitable for the region (Shih et al., 2007; Miao et al., 2018).

## 2 Materials and methods

### 2.1 Experimental site and materials

**Figure 1:**
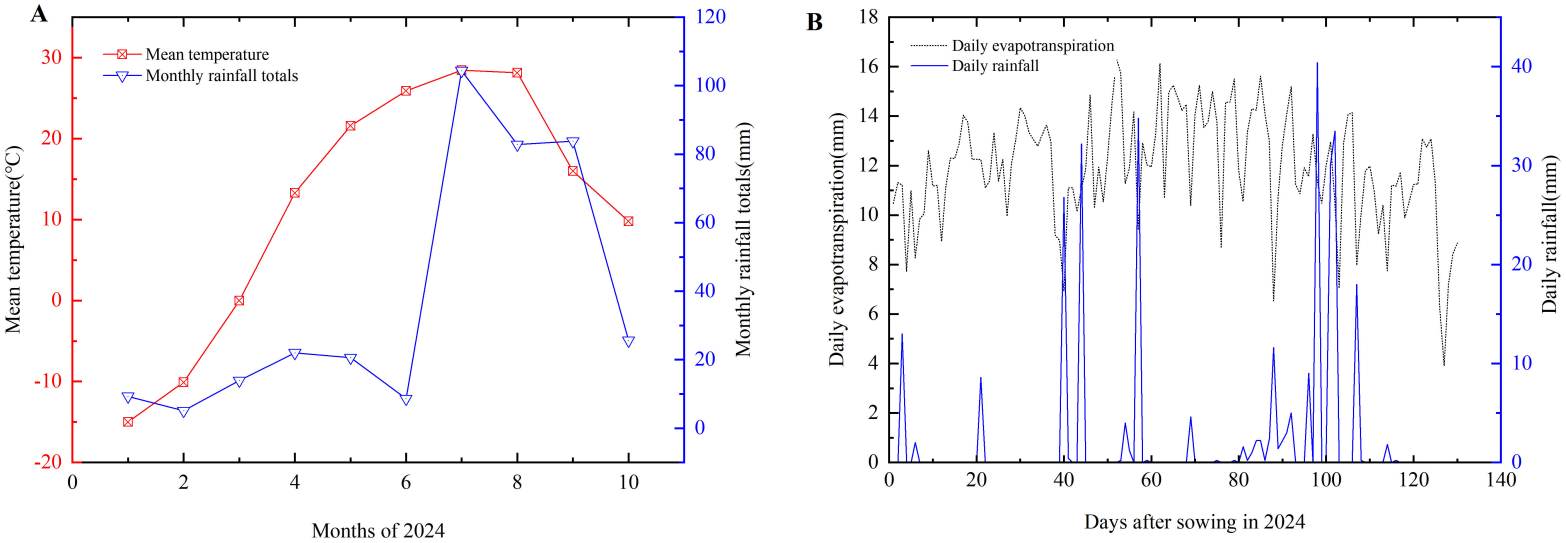
The monthly meteorological data **(A)** and daily evapotranspiration and rainfall **(B)** in 2024.

The experiment was conducted from May to September 2024 at Huaxing Farm in Changji City, Xinjiang (44.22°N, 87.29°E, 31m above sea level). The study area is characterized by a temperate continental arid climate, with an average annual rainfall of 190 mm, ≥10°C accumulated temperature of 3,450°C, annual sunshine duration of 2,700 hours, and a frost-free period of 160–190 days. The annual total ET° is approximately 900-1100 mm, with the summer months (June to August) accounting for more than 60% of the annual total. The interannual variation of precipitation was not significant and the distribution of precipitation during the planting season was relatively even in Changji city and northern Xinjiang region. Comparison with climate data from the past decade shows that the climatic characteristics during the experimental year were within normal fluctuation ranges without significant anomalies. The experimental soil is classified as sandy loam, with a bulk density of 1.39 g/cm^3^ at 0–20cm depth. The soil has a field capacity of 18.2% and nutrient contents of 78.53 mg/kg for nitrogen, 71.71 mg/kg for phosphorus, and 192.07 mg/kg for potassium. The pH of the soil is 7.06. Meteorological data for the experimental site are illustrated in **Figure 1**.

The corn variety used in the field experiment was Xinyu 74, which is renowned for its strong germination capability under drought conditions, robust seedling growth, vigorous vegetative development, and semi-compact mature plant structure. Particularly noteworthy is Xinyu 74’s outstanding drought resistance, enabling it to maintain stable yield performance even under water stress conditions.

### 2.2 Experimental design

This study established four irrigation treatments based on the CK (the control group, local irrigation volume with full irrigation). The CK irrigation regime, following local agricultural protocols integrating empirical field data and regional governmental advisories, was based on field capacity-based full irrigation requirements. The water supply during emergence was constant across all treatments, while the remaining treatments implemented water conservation from the seedling stage. Among these treatments, W1 represented mild RDI (10% irrigation volume reduction compared to CK), W2 represented moderate RDI (20% reduction compared to CK), W3 represented severe RDI (30% reduction compared to CK), and W4 represented extremely severe RDI (40% reduction compared to CK). **Table 1** presents the specific irrigation amounts for each growth stage of maize across the whole treatment period. The irrigation timing and fertilization regime were strictly consistent with conventional farm practices in the local area.

**Table 1:** Irrigation amounts of maize during the whole growth period under different treatments.

Irrigation Amount/(m^3^·ha^−1^)							
Treatments	Seedling water	Sowing- Jointing stage	Jointing-Heading stage	Heading-Silking stage	Silking-Filling stage	Filling-Maturity stage	Total Irrigation Amount
CK	750	900	1350	540	630	630	4800
W1	750	810	1215	486	567	567	4395
W2	750	720	1080	432	504	504	3990
W3	750	630	945	378	441	441	3585
W4	750	540	810	324	378	378	3180

**Figure 2:**
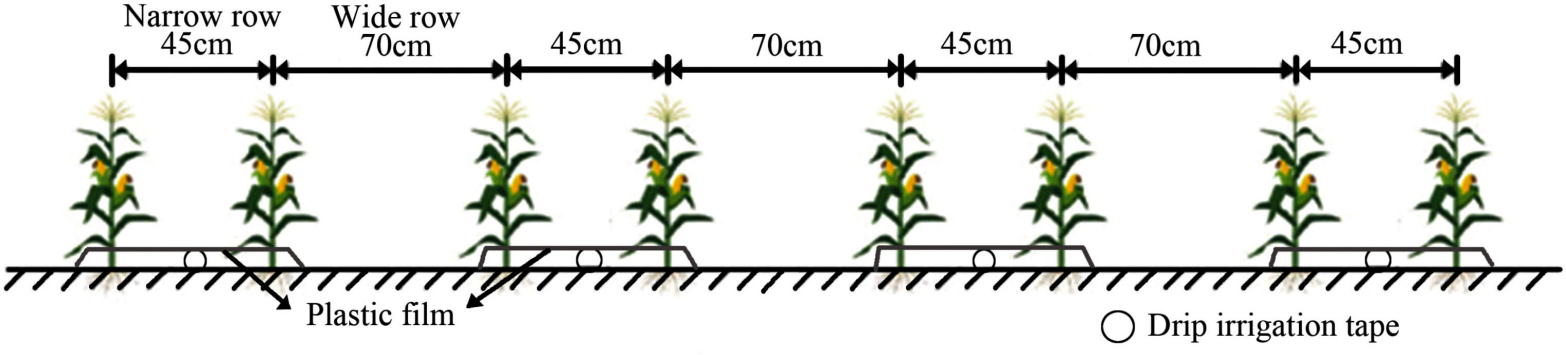
Schematic diagram of the maize planting pattern.

Maize was sown on May 24, 2024, and harvested on September 27, 2024. Similar to the local conventional planting pattern, the experiment employed drip irrigation under plastic mulch (**Figure 2**) and followed a wide-narrow row configuration of 70 cm for the wide rows and 45 cm for the narrow rows. The plants were spaced 20 cm apart, resulting in a planting density of 6,000 plants per mu.

**Figure 3:**
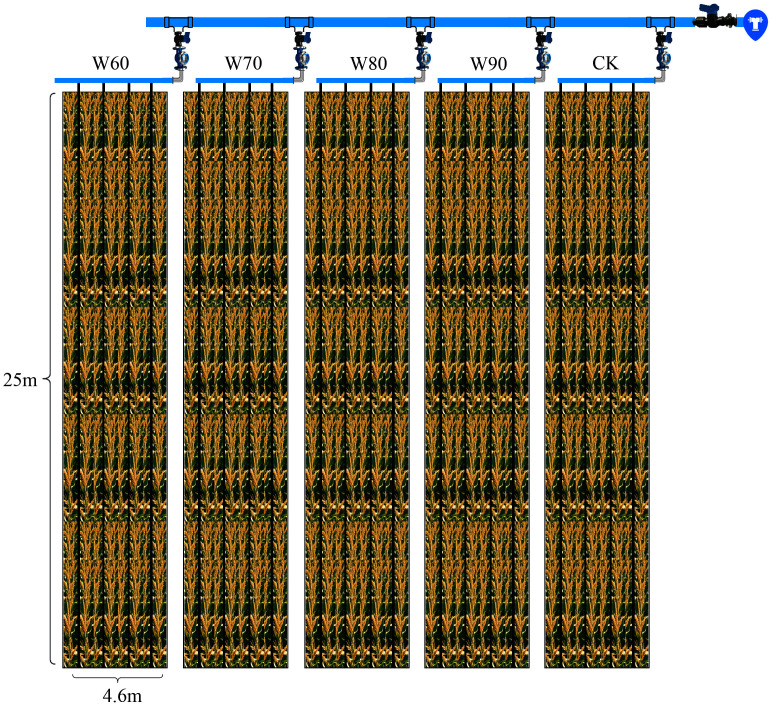
Schematic diagram of the maize experimental plot.

All experimental plots measured 25 meters in length and 4.6 meters in width, with a 70-centimeter isolation ridge constructed between the plots to prevent lateral water infiltration. Each plot was divided into three sampling zones—front, middle, and rear—each measuring 7 meters in length and 3 meters in width. A schematic diagram of the experimental plot layout is provided in **Figure 3**.

### 2.3 Measurement of maize growth and developmental traits under RDI

Five maize plants with uniform growth were selected and labeled for periodic measurement of growth and developmental traits. These traits included plant height, which was measured as the vertical distance from the soil surface to the highest point of the uppermost leaf before tasseling and to the tip of the central stem tassel after tasseling. The stem diameter was measured at the flattened, wide surface of the third internode above ground. The Leaf Area Index (LAI) was also determined using the punch method.

The LAI was determined as follows: Four plants were randomly selected from each plot during each growth stage, and all leaves were excised. A 1 cm diameter punch was used to sample leaf discs separated from the remaining leaf tissue. To account for potential variations in the area-to-weight ratio across the lamina, discs were systematically taken from the middle region of each leaf (between the midrib and margin, avoiding major veins). The punched discs and the residual leaf material were placed in separate bags and oven-dried to a constant weight. The LAI was subsequently calculated using (Equations 1, 2).


(1) $$\begin{array}{c} \text{Leaf area}=\text{Number of leaf punches}\times \text{Area of a single punch}/\\ \text{Dry weight of punched leaf discs}\times \\ \text{Dry weight of punched leaf discs + }\\ \text{ Dry weight of residual leaf tissue}) \end{array}$$

(2) LAI=Total leaf area/Ground area

### 2.4 Biomass under RDI across growth stages

Four representative maize plants were randomly selected from each treatment plot at each growth stage. The plants were then separated into their parts: stems, leaves, tassels, and ears. Initially, the samples were de-enzyme at 105°C for 30 minutes, then oven-dried at 75°C until they reached a constant weight. The dry matter weight of each organ was recorded.

### 2.5 Determination of stress resistance indices in maize under RDI conditions

At each growth stage (jointing stage, bell-mouthed stage, tasseling stage, filling stage, milk-ripe stage, waxy-ripe stage), approximately 7 days after irrigation treatment, five representative plants were randomly selected from each plot between 9:30 and 11:30 AM on clear, cloudless mornings for chlorophyll fluorescence measurements. Functional leaves were simultaneously collected from each treatment for physiological assays, including the determination of superoxide dismutase (SOD) activity using the nitroblue tetrazolium (NBT) photoreduction method, malondialdehyde (MDA) content via the thiobarbituric acid (TBA) method, peroxidase (POD) activity with the guaiacol colorimetric method, proline (Pro) content using the ninhydrin colorimetric method, chlorophyll content by spectrophotometry, and superoxide anion radical (O_2_^-^) content according to the hydroxylamine oxidation method (Gao, 2012).

### 2.6 Maize yield under RDI conditions

During harvest time, the yield was measured for the entire plot, excluding the border rows. Twenty maize plants were randomly sampled from each experimental plot. Before threshing, measurements were taken for ear length, ear diameter, number of ears, and kernel rows per ear. After threshing, the weight of 1,000 kernels was determined, and grain moisture content was measured using a calibrated grain moisture tester (PM-8188). The actual yield was calculated based on the national grain moisture standard of 14.0%.

### 2.7 Quality assessment of maize under RDI treatments

After measuring the yield, maize kernels from each treatment were thoroughly mixed and sent to the Shandong Academy of Agricultural Sciences for quality analysis. The analyzed parameters included protein, starch, amylose, fat, and ash content. Each parameter was evaluated in duplicate, and the average value was calculated for final reporting.

### 2.8 Data processing and statistical analysis

The data were initially processed using Microsoft Excel 2021 for fundamental analysis. Subsequently, a one-way analysis of variance (ANOVA) was performed using SPSS 27 to assess the significance of differences in specific measured parameters across the various treatments. Where ANOVA indicated significant differences (p< 0.05), *post-hoc* comparisons were performed using Tukey’s honest significant difference (HSD) test to identify specific treatment differences. Graphical representations of the data were created using Origin 2022.

Finally, the TOPSIS-entropy weight method was used to comprehensively evaluate the various indicators by calculating the information entropy and determining the weights of each indicator. The process involved several steps: (1) constructing and normalizing the original data matrix; (2) calculating the information entropy for each indicator; (3) determining the information utility values and their respective weights; and (4) computing the distances of each treatment to the positive and negative ideal solutions. This process ultimately led to the derivation of a comprehensive evaluation index, which helped identify the optimal irrigation strategy.

## 3 Results

### 3.1 Effects of different RDI treatments on maize plant height and stem diameter

**Figure 4:**
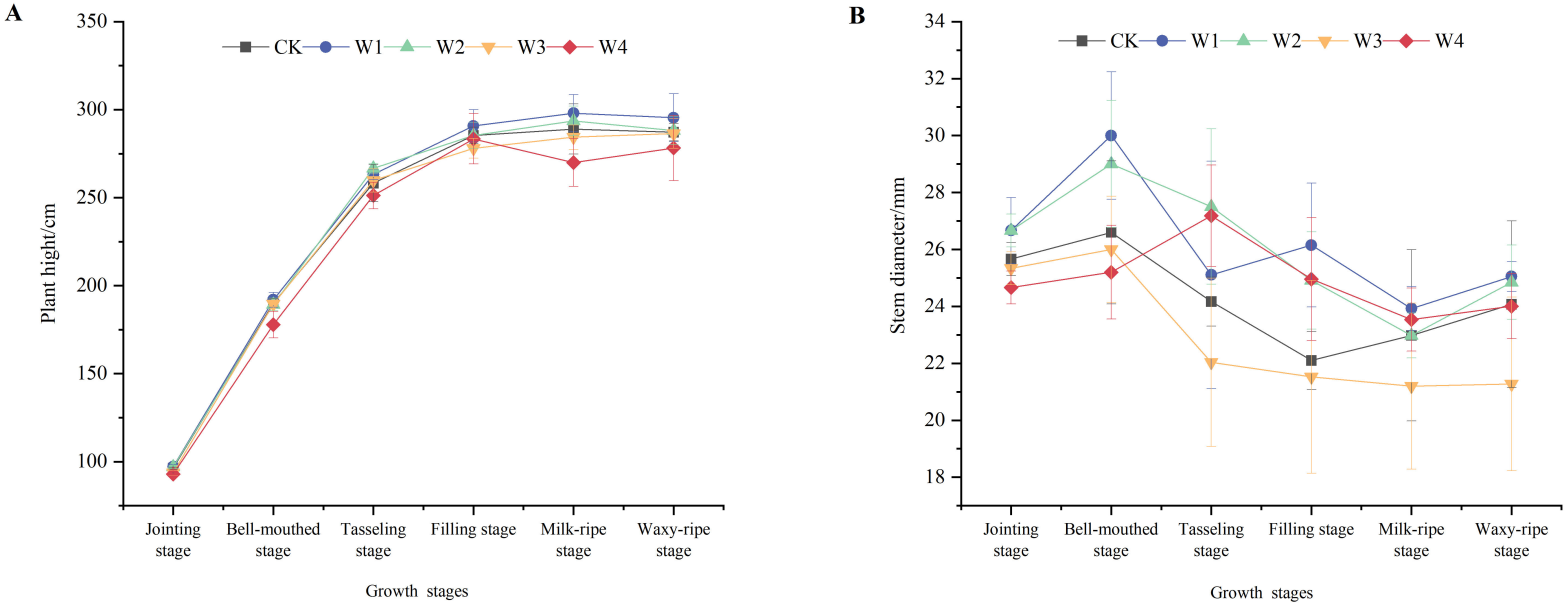
Changes in the plant height **(A)** and stem diameter **(B)** of maize plants under different irrigation treatments.

As shown in **Figure 4**, plant height in all treatment groups increased rapidly before the tasseling stage, after which the growth rate slowed and eventually stabilized. The stem diameter increased before the bell-mouthed stage, then showed a decreasing trend until the milk-ripe stage, and gradually stabilized after that. The W1 treatment exhibited superior plant height and stem diameter from the bell-mouthed to filling stages compared to other treatments, indicating that mild RDI did not significantly hinder the development of vegetative organs in maize plants.

### 3.2 Effects of different RDI treatments on LAI and biomass

**Figure 5:**
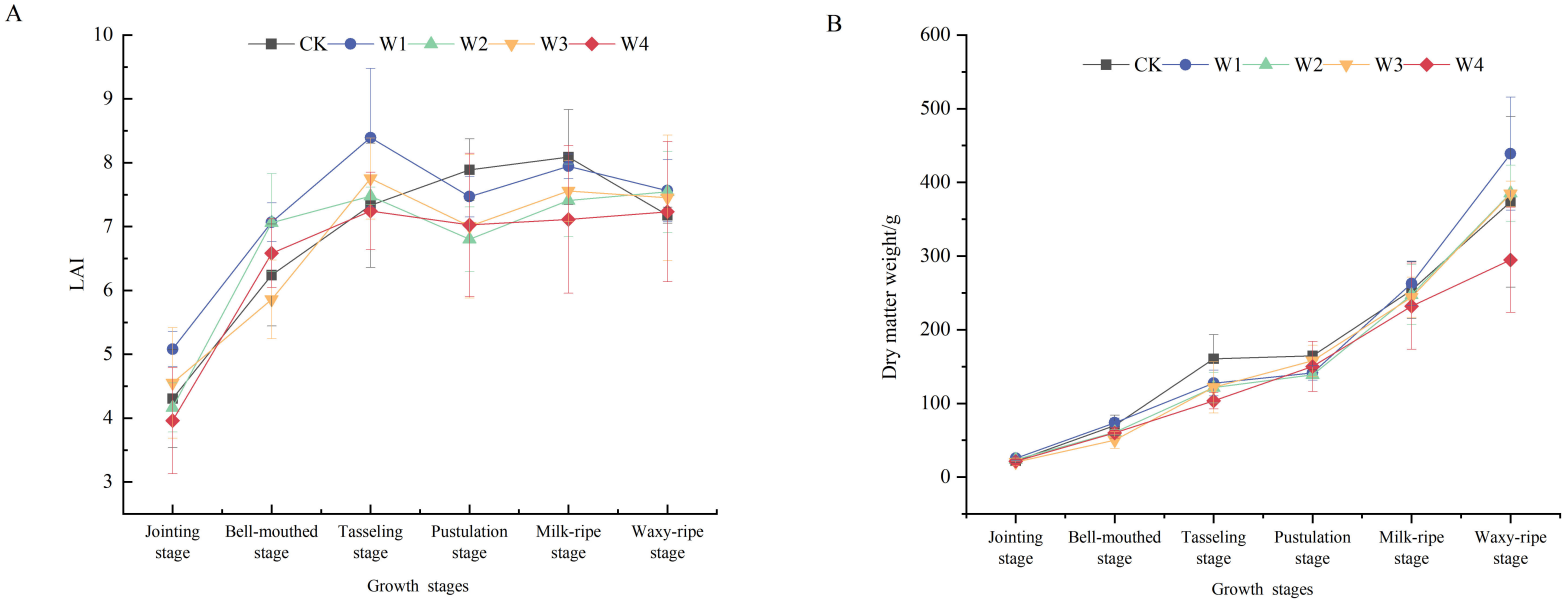
Changes in the LAI **(A)** and dry matter weight **(B)** of maize under different irrigation treatments.

Overall, the LAI of all treatment groups increased rapidly from the bell-mouthed stage to the tasseling stage, reaching a peak, and then slightly declined or remained stable during the filling stage (**Figure 5**). Throughout the growth cycle, maize under the W1 treatment maintained a relatively higher LAI, particularly during the tasseling and filling stages, indicating superior growth performance. In contrast, the W4 treatment exhibited lower LAI values at all growth stages, suggesting that extreme RDI negatively affected maize growth.

The accumulation of dry matter in crops is crucial for the biomass and final economic yields. The relationship between LAI and dry matter accumulation was examined through correlation analysis across different growth stages. A significant positive correlation was observed between these variables (*r* = 0.62, *p*< 0.01), indicating that a higher LAI was associated with greater assimilate accumulation. During the early vegetative growth stages, dry matter accumulation was relatively slow, with minimal treatment differences. However, the accumulation rate increased significantly during the later reproductive growth stages. The highest dry matter accumulation occurred at the waxy-ripe stage, with the W1 treatment showing the obvious accumulation, 19.5% higher than the CK. Conversely, the W4 treatment exhibited the lowest accumulation, 21.1% lower than CK.

### 3.3 Effects of different RDI treatments on chlorophyll content and fluorescence parameters in maize leaves

**Figure 6:**
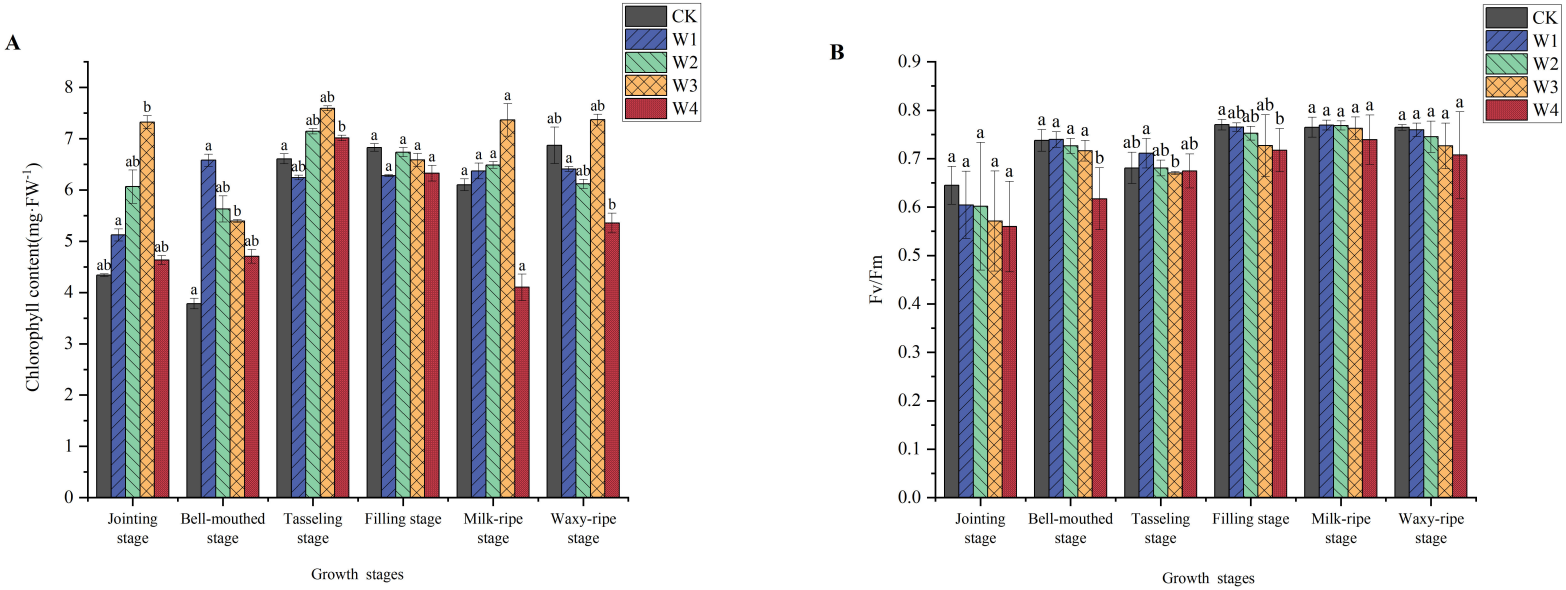
Changes in the chlorophyll content **(A)** and fluorescence **(B)** of maize under different irrigation treatments (different letters in the figure indicate significant differences between treatments at *p*< 0.05).

The chlorophyll content exhibited a distinct trend in maize during its growth stages (**Figure 6**). From the jointing stage to the tasseling stage, chlorophyll content increased, correlated with the vigorous vegetative growth phase. However, from the tasseling stage to the waxy-ripe stage, there was a decline in chlorophyll content, likely due to leaf senescence. RDI treatments notably increased chlorophyll content during the early growth stages. During the reproductive growth stage, the chlorophyll content in W1, W2, and W3 treatments showed a slight decrease, while W4 exhibited a significant decrease. Overall, the W2 and W3 treatments maintained relatively higher chlorophyll content in each period.

The variation in chlorophyll fluorescence parameters (Fv/Fm) indicated fluctuations under different treatments, but the overall trend was not significant. Comparatively, the CK group maintained higher and more stable Fv/Fm values. The Fv/Fm values of the W1, W2, and W3 treatments showed some fluctuations. However, they remained close to the CK groups, and these differences all fell within the normal range for healthy maize.

### 3.4 Effects of different RDI treatments on proline content in maize leaves

**Figure 7:**
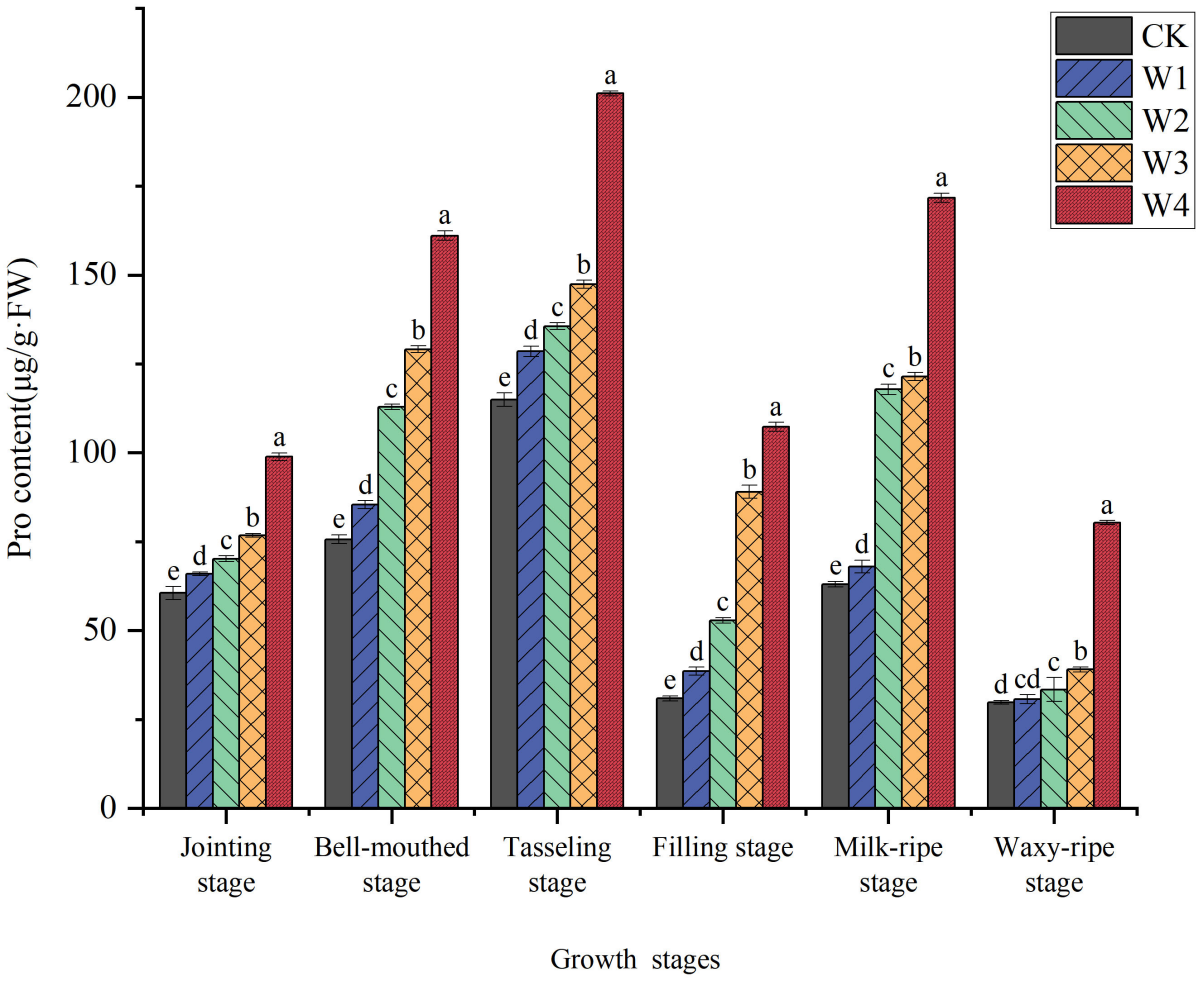
Changes in the Pro content of maize leaves under different irrigation treatments (different letters in the figure indicate significant differences between treatments at *p*< 0.05).

As illustrated in **Figure 7**, proline content in maize leaves significantly increased under RDI treatments. At the tasseling stage, proline accumulation in all treatment groups reached peak levels, showing increases of 11.80%, 17.97%, 28.20%, and 74.90% compared to the control (CK), respectively. The W1 treatment exhibited relatively lower proline content, indicating that mild RDI did not impose significant stress on maize and almost met its water requirements. The moderate Pro accumulation in W2 and W3 suggests sub-threshold stress levels, where maize plants likely activated osmotic adjustment mechanisms without severe growth compromise. In contrast, the W4 treatment showed consistently higher proline content during all stages, suggesting that this treatment imposed a higher level of water stress on maize and induced the proline production to perform infiltration adjustment in cells. Moreover, the final yield data demonstrate a correlation with proline content.

### 3.5 Effects of different RDI treatments on MDA content in maize leaves

**Figure 8:**
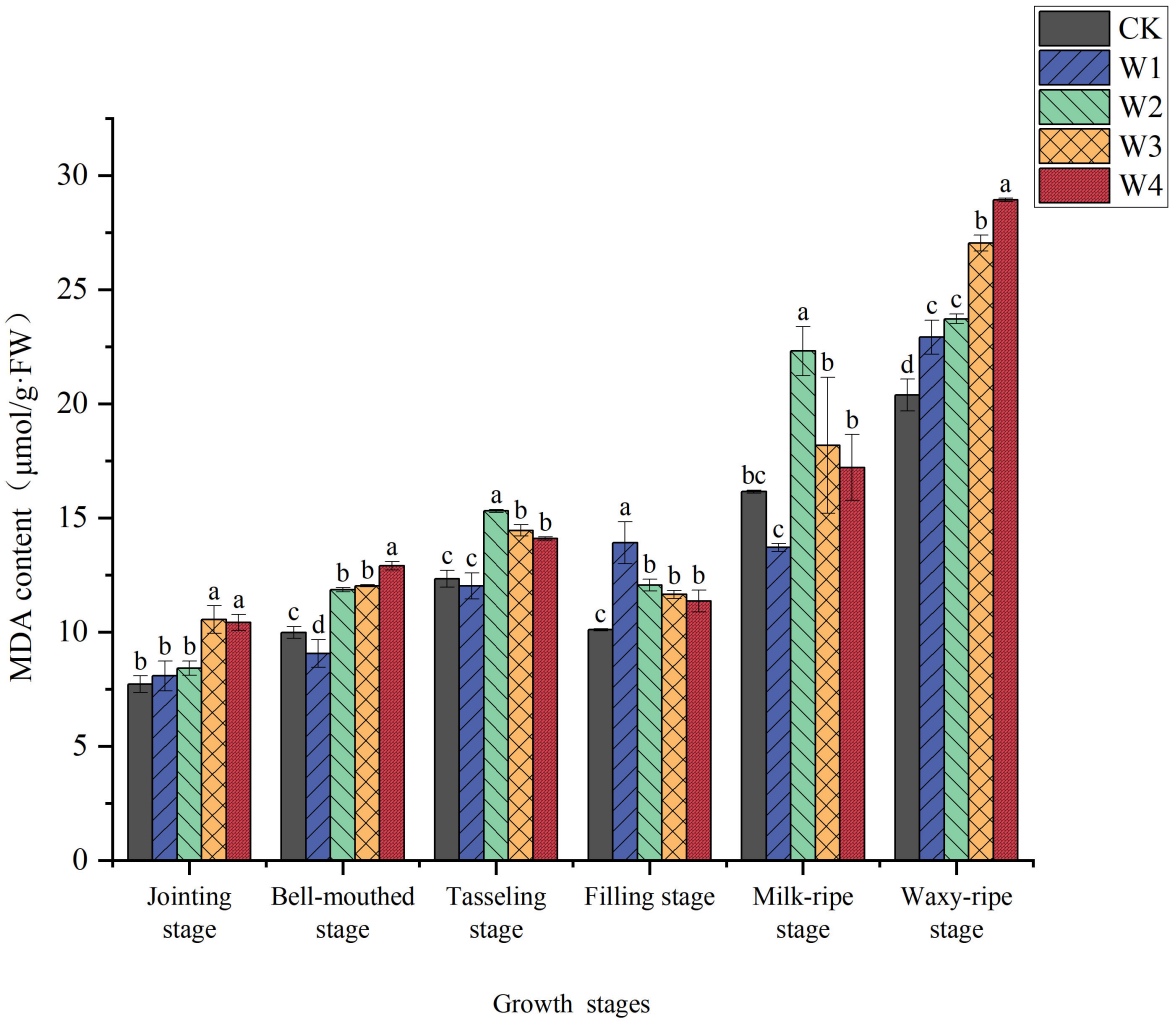
Changes in the MDA content of maize leaves under different irrigation treatments (different letters in the figure indicate significant differences between treatments at *p*< 0.05).

As shown in **Figure 8**, the MDA content in maize leaves exhibited significant differences under different irrigation treatments. The W1 treatment showed relatively lower MDA content, indicating that mild RDI did not cause significant damage to maize leaves. In contrast, the W4 treatment exhibited higher MDA content during multiple growth stages, suggesting that extreme RDI caused substantial oxidative damage to leaves cells. Among all treatments, MDA content reached its highest point at the waxy-ripe stage, rising by 12.4%, 16.4%, 32.7%, and 41.9% compared to CK. This obvious accumulation can be attributed to the waxy-ripe stage represents a critical period of water sensitivity in maize, where cellular metabolic activity and resource translocation requirements are particularly high; and the prolonged exposure to water deficit treatments resulted in cumulative oxidative stress, leading to enhanced production of reactive oxygen species that ultimately triggered more severe membrane damage during this vulnerable developmental phase.

### 3.6 Effects of different RDI treatments on reactive oxygen species metabolism in maize leaves

**Figure 9:**
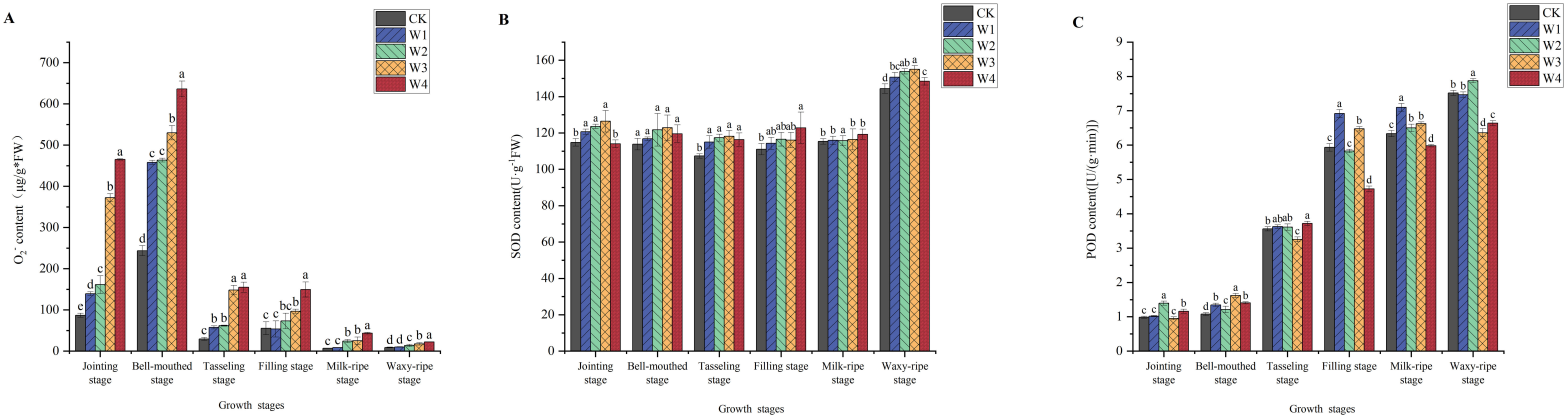
Changes in O_2_^-^ **(A)**, SOD **(B)**, and POD **(C)** in maize leaves under different irrigation treatments (different letters in the figure indicate significant differences between treatments at *p*< 0.05).

As shown in **Figure 9**, different irrigation treatments significantly influenced the O_2_^-^ content and the activities of antioxidant enzymes (SOD and POD) in maize leaves. From the seedling stage to the tasseling stage, the O_2_^-^ content remained relatively high, exhibiting significant differences among treatments. At the jointing stage, the O_2_^-^content in the different treatments increased by 60%, 85%, 328%, and 435% compared to the CK. In response, maize plants increased their SOD and POD activities to enhance antioxidant capacity and reduce the damage caused by ROS. As the intensity of water-saving measures increased, SOD and POD activities gradually rose. The W1 and W2 treatments showed significantly higher enzyme activities than CK. However, the W4 treatment exhibited lower than CK in antioxidant enzyme activities.

### 3.7 Effects of different RDI treatments on maize yield and quality

There were no significant differences in kernels per ear among the irrigation treatments (**Table 2**). However, W1 treatment resulted in the highest number of kernels per ear, indicating a positive effect on yield formation to some extent. The W1 treatment showed improvements across various parameters, with a significant increase in final yield by 8.0% compared to the CK. In contrast, other RDI treatments experienced yield reductions of 4.0%, 5.4%, and 6.5%, respectively, but the reductions were not statistically significant (*p* = 0.057). Despite the implementation of deficit irrigation, the overall water deficit was not severe, which helped prevent significant yield losses.

**Table 2:** Maize yield and its constituent elements under different irrigation treatments.

Treatments	Ear length (cm)	Ear Diameter (cm)	Ear weight (g)	grain number per ear	1000-Grain Weight (g)	Theoretical Yield (kg·ha^−2^)
CK	17.97 ± 1.77a	5.07 ± 0.32b	228.51 ± 41.05a	508.15 ± 101.87a	384.96 ± 0.97ab	16322.7 ± 506.55b
W1	18.35 ± 2.48a	5.41 ± 0.31a	240.12 ± 57.51a	540.65 ± 107.08a	388.71 ± 10.59a	17631.3 ± 484.95a
W2	17.65 ± 1.89a	5.11 ± 0.37b	229.62 ± 43.83a	499.50 ± 87.09a	375.78 ± 5.61ab	15668.85 ± 485.1b
W3	17.92 ± 2.30a	5.26 ± 0.43ab	224.06 ± 50.67a	496.90 ± 92.18a	371.20 ± 13.22bc	15442.2 ± 485.25b
W4	18.69 ± 1.72a	5.31 ± 0.33ab	219.05 ± 30.64a	510.70 ± 68.39a	357.62 ± 8.02c	15258.45 ± 485.4b

In terms of quality, all RDI treatments improved the quality of maize kernels to some extent (**Table 3**). The ash content remained unchanged under treatments, while fat content increased by 6.7% in the W1 treatment and decreased by 3.4% in the W3 treatment. The other three quality indicators (protein, starch, and amylose content) were higher in all treatments compared to the CK. This indicated that appropriate irrigation management might enhance the quality of maize kernels. Under commercial production scales, although the compositional changes in individual kernels are modest, their aggregate cumulative effects can significantly enhance the economic value of the end products.

**Table 3:** Maize grain quality under different irrigation treatments.

Test items					
Treatments	Protein (g/100g)	Fat (g/100g)	Ash (g/100g)	Starch (g/100g)	Amylose/%
CK	7.47	3.0	1.2	67.7	15.55
W1	7.59	3.2	1.2	68.1	17.30
W2	7.74	3.0	1.2	68.6	17.31
W3	7.85	2.9	1.2	68.4	17.16
W4	7.51	3.0	1.2	67.8	17.13

Overall, W1 not only prevented yield loss but also supported yield formation and improved quality. This emphasizes the significant value of W1 as a water-saving irrigation method, highlighting the potential of optimized deficit irrigation strategies to balance crop productivity, quality, and water resource efficiency.

### 3.8 Comprehensive evaluation of different RDI treatments

This study used the TOPSIS-entropy weight method to analyze and evaluate maize agronomic traits, yield, and quality to identify the optimal water-saving strategy for maize growth and development. Based on the experimental data, the best water-saving measures for promoting high yield and quality in maize were identified. **Table 4** showed that the kernel number per ear and theoretical yield had the lowest information entropy values, at 0.64 and 0.644, respectively. This resulted in higher information utility values (0.36 and 0.356, respectively) and correspondingly higher weights (10.739 and 10.604, respectively). These factors were critical in the comprehensive evaluation, indicating that yield and kernel number per ear significantly impact the overall assessment in empirical studies. In the final evaluation results (**Table 5**), the mild water-saving treatment (W1) performed the best, achieving the highest comprehensive evaluation index of 0.728.

**Table 4:** Calculation of evaluation indicators and their corresponding TOPSIS entropy weight values.

Entropy Weight Method					
Classification	Item	Type of Indicator	Entropy	Information Utility Value	Weight (%)
Agricultural traits	Plant height	Positive indicators	0.781	0.219	6.541
	Stem diameter	Positive indicators	0.844	0.156	4.658
	LAI	Positive indicators	0.764	0.236	7.05
	Dry matter weight	Positive indicators	0.839	0.161	4.801
	Chlorophyll content	Positive indicators	0.845	0.155	4.626
Yield	Ear length	Positive indicators	0.773	0.227	6.773
	Ear Diameter	Positive indicators	0.754	0.246	7.334
	grain number per ear	Positive indicators	0.64	0.36	10.739
	1000-Grain Weight	Positive indicators	0.861	0.139	4.137
	Bare top length	Reverse indicators	0.813	0.187	5.561
	Theoretical Yield	Positive indicators	0.644	0.356	10.604
Quality	Protein	Positive indicators	0.716	0.284	8.459
	Fat	Positive indicators	0.772	0.228	6.797
	Starch	Positive indicators	0.739	0.261	7.766
	Amylose	Positive indicators	0.861	0.139	4.154

**Table 5:** The final results of the TOPSIS evaluation method.

Treatments	Positive Ideal Distance(D+)	Negative Ideal Distance(D-)	Comprehensive Score Index	Ranking
W1	0.31766367	0.84898545	0.72771276	1
W2	0.59481807	0.65983589	0.52591066	2
W3	0.64776809	0.5985263	0.48024472	3
W4	0.7948776	0.45360306	0.36332406	4
CK	0.75207377	0.42588587	0.36154538	5

## 4 Discussion

**Figure 10:**
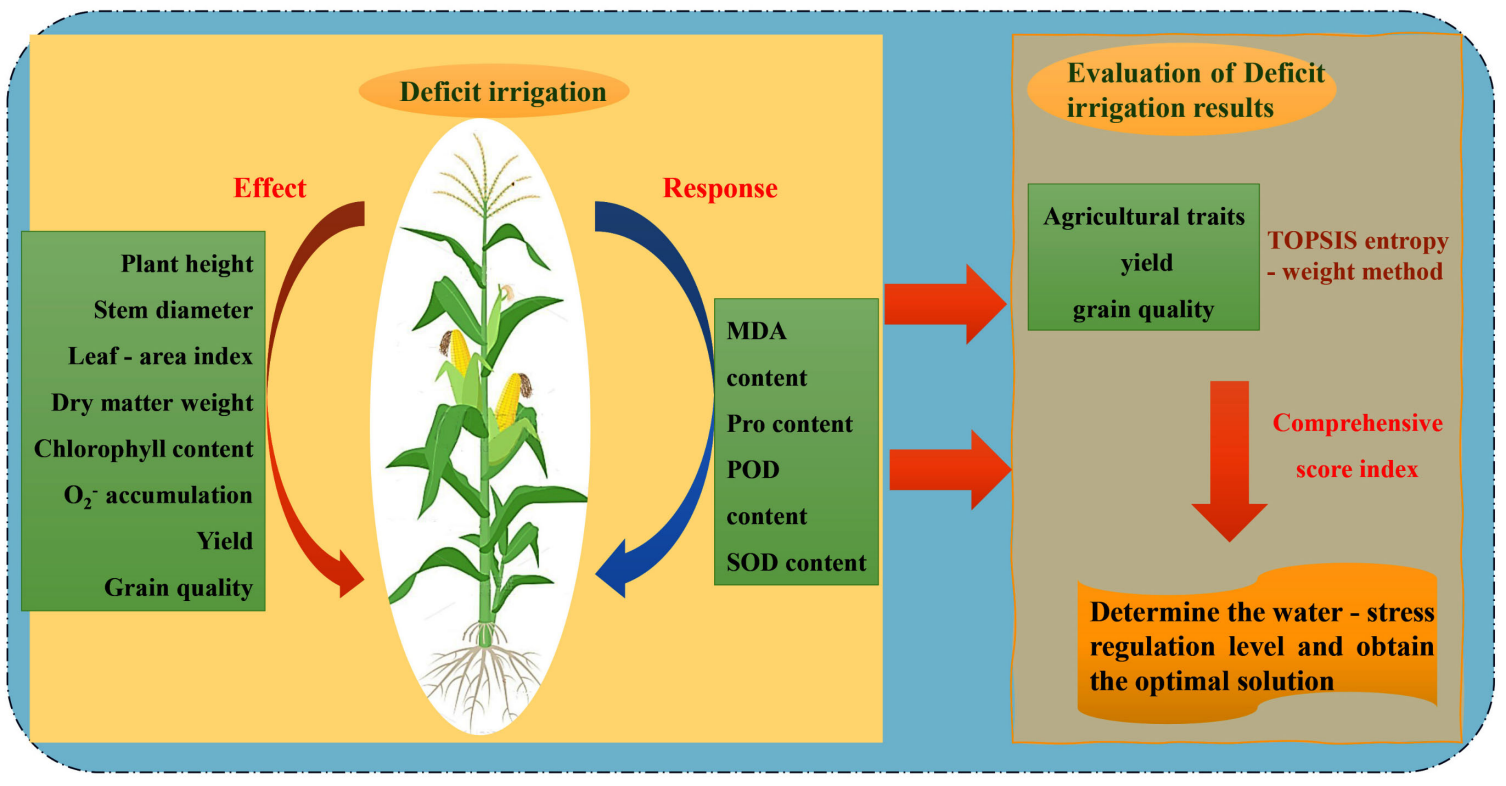
Technical roadmap of regulated deficit irrigation.

RDI is one of the widely used water-saving technologies and is crucial in promoting sustainable agricultural development. This study, conducted at Huaxing Farm in Xinjiang, examined the effects of various RDI treatments on agronomic characteristics, physiological-biochemical characteristics, yield, and quality in maize. Furthermore, the TOPSIS-entropy weight method was employed for a comprehensive analysis to provide precision irrigation of maize irrigation in this region, and in other arid and semi-arid areas (**Figure 10**). The comprehensive evaluation results obtained through the TOPSIS-entropy weight method (**Tables 4**, **5**) demonstrated strong consistency with the physiological responses. The W1 treatment achieved the highest score (0.728), which corresponds well with its optimized physiological status - effectively activating the antioxidant system while avoiding excessive energy consumption for osmotic adjustment. This balanced physiological state ultimately led to superior yield performance and quality improvement. Based on these results, an optimal irrigation strategy is tailored for Huaxing Farm and similar agro-climatic conditions.

The perspective of maize growth and development indicators, particularly plant height and stem diameter, provided quantifiable evidence of RDI-induced photoassimilate partitioning in maize (Poudel, 2023). The W1 group exhibited greater plant height after the filling stage and more stable stem diameter compared to other treatments, indicating optimized carbon allocation under mild RDI (**Figure 4**). This result was consistent with previous findings that suggested “moderate stress induces compensatory growth” (Li and Huang, 2001; Hu et al., 2010). The mechanism underlying this phenomenon was that mild RDI stimulated internal regulatory responses in maize, influencing the preferential allocation of carbon. As a result, more carbon allocation was transported to the tissues, promoting stem growth and lignification (Naikoo et al., 2019). The increased degree of lignification enhanced the mechanical support capacity of the stem, enabling it to withstand external factors better during the later growth stages and providing a stable foundation for overall plant growth (Fan, 2022). Moreover, mild RDI promoted maize root growth and enhanced mineral nutrient uptake capacity. According to the basis of deep roots giving rise to flourishing leaves, dry matter accumulation was ultimately altered (Fan et al., 2006; Ma et al., 2025). In contrast, the W4 treatment, which subjected the plants to a severe water deficit, reduced photosynthesis. This disruption decreased the synthesis and transport of photoassimilate, constraining compensatory nutrient foraging capacity under severe stress, which failed to meet their nutritional demands for growth. Consequently, plant height and stem diameter stagnated, and dry matter accumulation was hindered in W4 plants.

The variation in LAI further revealed phasic leaf development plasticity under differential water treatments. The W1 treatment exhibited higher LAI during the tasseling stages compared to other treatments, it was 12.63%, 10.85%, 7.63%, and 13.71% higher than those of other treatments (**Figure 5**). The result suggested that mild RDI enhanced maize leaf growth and development by modulating stomatal aperture dynamics and accelerating leaf expansion rates (Zhang et al., 2021). This moderate stress also promoted cell division processes and leaf expansion, facilitating sustained optimal growth under water-limited conditions. In contrast, the W4 treatment resulted in a lower LAI, suggesting that extreme RDI severely restricted leaf growth. This leaf growth limitation was likely due to reduced cell turgor pressure caused by water deficit, which adversely impacted leaf expansion rates and photosynthetic surface development. Consequently, these constraints reduced photosynthetic efficiency and dry matter accumulation under severe stress (Song et al., 2019; Széles et al., 2023).

Chlorophyll content and fluorescence parameters were intrinsically linked, collectively reflecting the photosynthetic performance. Overall, the W2 and W3 treatments exhibited relatively higher chlorophyll content in each period (**Figure 6**). This chlorophyll elevation might be attributed to the early-phase RDI, wherein maize upregulated pigment biosynthesis to optimize photosynthetic light capture, thereby compensating for water scarcity and sustaining photosynthetic efficiency for growth, development maintenance (Cai et al., 2020). However, under severe RDI (W4), exacerbated chlorophyll catabolism and premature leaf senescence in the later growth stages drove a marked depletion of photosynthetic pigments and severely impacted photosynthesis. So, chlorophyll content significantly declined (Tobiasz-Salach et al., 2023), and net photosynthetic rates and biomass allocation were suppressed. The stability of fluorescence parameters in W1, being close to CK (**Figure 6**), demonstrated that mild RDI could sustain photochemical efficiency and maintain the functional integrity of PSII reaction centers. Photosynthetic performances in maize were stabilized under water-limiting conditions, indicating that maize had strong adaptability and self-regulation capabilities at that time (Netondo et al., 2004; Efeoğlu et al., 2009; Luo et al., 2016). Under moderate water-deficit conditions, maize maintained optimal photosynthetic activity, enabling robust biomass accrual and reproductive success.

Maize plants respond to water stress by integrating both physiological and biochemical pathways, such as osmotic adjustment and the antioxidant system, to mitigate damage and maintain growth. Only a slight increase in proline content was observed under mild RDI (W1) (**Figure 7**). Meanwhile, it was accompanied by lower levels of MDA (**Figure 8**) and elevated activities of antioxidant enzymes (**Figure 9**). These results indicated that the W1 treatment did not induce severe osmotic stress in plants. In high-temperature conditions, osmotic stress may be exacerbated, potentially leading to alterations in proline accumulation and impairment of antioxidant enzyme activity. Instead, maize maintained cellular homeostasis by activating the antioxidant defense system, ensuring normal physiological metabolism. However, the findings from the W4 treatment revealed a significantly higher proline content in plants (**Figure 7**), suggesting that maize shifted from relying on antioxidant defenses to synthesizing and accumulating proline (**Figure 7**) to combat severe osmotic stress under extreme RDI (Hong et al., 2017) (Shabbir et al., 2020).

MDA content is associated with membrane lipid peroxidation and oxidative stress, and its variation reflects the extent of maize’s response to water stress (Morales and Munné-Bosch, 2019). The observation of relatively lower MDA content in the W1 treatment (**Figure 8**) indicated that oxidative damage was not generated in maize leaves under mild RDI, directly reflecting the effectiveness of maize’s antioxidant defenses in maintaining membrane integrity (Guo et al., 2018). In contrast, the W4 treatment exhibited higher MDA content during multiple growth stages, indicating that extreme RDI caused significant oxidative damage to maize. It likely resulted from the water deficit leading to the accumulation of ROS within cells, exceeding the scavenging capacity of the antioxidant system and resulting in intensified membrane lipid peroxidation (Song et al., 2012). Under water stress, plants regulated the levels of antioxidant enzymes (SOD and POD) to counteract the accumulation of O_2_^-^ (Sharma et al., 2012 ^;^ Guo et al., 2018 ^;^ Hussain et al., 2019). Corresponding to the trend of O_2_^-^ content, indicating that maize activated its antioxidant defense mechanisms by enhancing antioxidant enzyme activities to scavenge excess O_2_^-^, reduce oxidative damage, and maintain the cellular redox balance (Wang et al., 2023).

The aforementioned growth, development, and physiological and biochemical changes in plants influence the yield and quality of crops. The W1 treatment produced not only a significant increase in yield but also improved grain quality (**Table 2**-3). This was likely due to the implementation of mild RDI, which optimized photoassimilate partitioning toward reproductive sinks, ensuring sufficient carbohydrate provision for optimal grain development. Moderate water deficit combined with optimal fertilization enhanced nitrogen (N), phosphorus (P), and potassium (K) uptake and utilization in maize, leading to improved grain quality (Yan et al., 2020). Additionally, the efficient operation of the antioxidant system effectively scavenges ROS within cells, thereby preventing oxidative impairment to grain morphogenesis and maintaining consistent grain filling dynamics (Yousaf et al., 2022; Vadez et al., 2024). Furthermore, mild RDI might alter metabolic pathways within maize plants by activating gene expression related to starch synthesis and protein metabolism. This process promoted the production of important quality components, such as starch and protein, thus enhancing the quality of maize kernels (Zhi-Meng et al., 2009; Wei and He, 2022; Han et al., 2025). Although other treatments did not show significant yield reductions, water stress still exerted subcritical perturbations, which impaired the grain-filling process.

Real-time data on the growth and development of maize plants was obtained through field experiments under different water treatments this year. Water-saving irrigation amount and the reasons for the high-quality and high-yield of maize under optimized irrigation were analyzed. The results indicated that mild RDI (W1) induced multifaceted improvements in growth and development, physiological and biochemical response, yield, and quality formation in maize, while extreme RDI (W4) produced relatively adverse effects. This provided a theoretical basis for sustainable irrigation strategies in arid and semi-arid regions, helping to develop precise irrigation management regimes that reconcile high-yield quality and efficient water-resource utilization. This study was conducted over a single growing season, but the experimental data still have certain scientific value. For example, the experimental year’s weather patterns showed no statistically significant anomalies with the past decade, and data from the untreated CK indicated minimal variation in both vegetative development and crop yield compared to those from the previous two years. However, the field experiment of a single growing season may introduce several limitations. The variations in precipitation patterns and total amounts across different years could result in differences in soil water dynamics, affecting maize’s response to various water treatments. Expanding research should further explore the effects of RDI on maize across diverse climatic conditions and multiple growing seasons. Conducting multi-year replicated trials, investigating diverse soil textures and land types, and performing cultivar-specific validation experiments. Additionally, investigating the long-term effects of water stress on both the soil environment and maize growth will provide more comprehensive scientific guidance for sustainable, climate-resilient agricultural systems.

## 5 Conclusion

As the research background on the climate, drought and irrigation-water scarcity at Huaxing Farm in Xinjiang, this study conducted field experiments on maize planting under different RDI treatments. This approach was critical for balancing efficient water conservation with agricultural productivity demand. The research through multiple dimensions of RDI on maize through multiple dimensions, including agronomic traits, physiological-biochemical characteristics, yield, and quality characters, provided a holistic understanding of how water deficit impacted maize planting. The TOPSIS-entropy weight method was employed for comprehensive evaluation. The conclusions were as follows: Different levels of RDI had varying effects on maize plants. Mild RDI (W1) promoted maize growth, increased yield, and improved quality to some extent, while extreme RDI (W4) significantly inhibited these aspects. The study identified mild RDI (W1) as the optimal irrigation strategy for maize planting in this region. The findings provide a foundation for adaptive irrigation strategies in water-limited maize systems for implementing water-saving irrigation of maize in arid and semi-arid areas. Further research is also necessary to enhance the theoretical framework and technological systems related to water-saving irrigation for maize planting.

## Data Availability

The original contributions presented in the study are included in the article/supplementary material. Further inquiries can be directed to the corresponding author.
